# Naringina, Trimetazidina e Barorreflexo na Lesão de Isquemia e Reperfusão Renal

**DOI:** 10.36660/abc.20210453

**Published:** 2021-08-09

**Affiliations:** 1 Universidade Estadual Paulista BotucatuSP Brasil Universidade Estadual Paulista, Botucatu, SP - Brasil.

**Keywords:** Flavanonas, Flavonóides, Trimetazidina, Vasodilatadores, Barorreflexo, Hipertensão, Insuficiência Cardíaca, Reperfusão Renal, Estresse Oxidativo

O artigo “Naringina e trimetazidina melhoram a sensibilidade do barorreflexo e a atividade elétrica do núcleo do trato solitário na lesão de isquemia e reperfusão renal”, publicado nesta revista,^1^ encontrou melhora na sensibilidade do barorreflexo em um modelo de lesão renal aguda de isquemia/reperfusão em ratos tratados com naringina e/ou trimetazidina.

A trimetazidina e a naringina são substâncias capazes de reduzir o estresse oxidativo, documentado em diversas situações. A trimetazidina é um fármaco com propriedades anti-isquêmicas, que atua diretamente na mitocôndria e é capaz de diminuir o estresse oxidativo. A naringina é um polifenol com propriedades antioxidantes presente em diversas frutas cítricas.

O estresse oxidativo está envolvido em diversos processos fisiopatológicos.^2^ Por outro lado, o distúrbio do reflexo barorreceptor está envolvido na patogênese da hipertensão^3^^,^^4^ e da insuficiência cardíaca.^5^ Além disso, esse distúrbio atua na doença renal crônica^6^ e na lesão renal aguda.^7^ O núcleo do trato solitário desempenha um papel fundamental na integração do reflexo barorreceptor e sua ação é influenciada pelo estresse oxidativo.^7^

A atenuação do barorreflexo na lesão renal aguda pode dificultar a resposta à instabilidade hemodinâmica durante um episódio de lesão renal aguda.^8^^,^^9^ A lesão renal aguda também mostra um aumento no estresse oxidativo.^2^ Radicais livres e espécies reativas de oxigênio são produzidos em abundância no dano renal devido à isquemia/reperfusão e inundação do sistema circulatório causando efeitos indesejáveis em diversos órgãos, incluindo o trato solitário, que, como vimos, é um importante integrador da atividade barorreflexa.

Em outros modelos, exceto na lesão renal aguda, o estresse oxidativo está correlacionado com a disfunção dos barorreceptores, sendo que os antioxidantes apresentaram melhorar em sua função. No entanto, na lesão renal aguda, é controverso se esse aumento do estresse oxidativo tem uma relação de causa e efeito com a atenuação do barorreflexo. Assim, se, em modelos de lesão renal aguda, ao bloquear o estresse oxidativo, pudéssemos restaurar o barorreflexo, seria evidente que o estresse oxidativo desempenhe esse papel.

A variação da relação entre a frequência cardíaca e a pressão arterial média em comparação ao desafio com a fenilefrina foi o índice barorreflexo realizado no estudo de Amini et al.,^1^ Utilizando uma técnica estereotáxica, antes de induzir a lesão renal aguda, implantou-se um eletrodo no núcleo do trato solitário dos ratos com atividade nuclear.^1^ Assim, além da melhora do barorreflexo, houve reversão da atenuação da atividade do núcleo do trato solitário documentados concomitantemente com a lesão de reperfusão.

Esses resultados têm implicações fisiopatológicas, na medida em que demonstram a participação do estresse oxidativo na disfunção do núcleo do trato solitário e consequentemente do barorreflexo na lesão renal aguda, bem como implicações terapêuticas, uma vez que incentiva o trabalho com esses fármacos no sentido de mitigar as complicações da lesão renal aguda em humanos,^9^^-^^11^ uma situação clínica que denota um prognóstico nefasto.^12^ Portanto, esta linha de pesquisa pode ajudar a compreender o tratamento em humanos com essa entidade nosológica. É importante notar que há evidências de profilaxia da nefropatia por contraste com trimetazidina em humanos.^13^^,^^14^
