# Supplementary material for: Naringina, Trimetazidina e Barorreflexo na Lesão de Isquemia e Reperfusão Renal
Source: Arq Bras Cardiol. 2021 Aug 9;117(2):298–9. [Article in Portuguese] doi: 10.36660/abc.20210453 (PMC8395796; doi:10.36660/abc.20210453)
Supplement: Translation

# Translation: Naringin, Trimetazidine and Baroreflex in Renal Ischemia-Reperfusion Injury

**Affiliations:** 2 BotucatuSP Brazil Universidade Estadual Paulista, Botucatu, SP - Brazil.

**Keywords:** Flavanones, Flavonoids, Trimetazidine, Vasodilatador Agents, Hypertension, Heart Failure, Renal Reperfusion, Oxidative Stress

The paper “Naringin and trimetazidine improve baroreflex sensitivity and electrical activity of solitary tract nucleus in renal ischemia-reperfusion injury,” published in this journal,^1^ found improvement in baroreflex sensitivity in an acute ischemia/reperfusion kidney injury model in rats treated by naringin and/or trimetazidine.

Trimetazidine and naringin are substances capable of reducing oxidative stress, documented in many situations. Trimetazidine is a drug with anti-ischemic properties, which acts directly on mitochondria and is able to decrease oxidative stress. Naringin is a polyphenol with antioxidant properties present in several citrus fruits.

Oxidative stress is involved in multiple pathophysiological processes.^2^ On the other hand, disorder of the baroceptor reflex is involved in the pathogenesis of hypertension^3^^,^^4^ and heart failure.^5^ Moreover, this disorder plays a role in chronic kidney disease^6^ and in acute kidney injury.^7^ The solitary tract nucleus plays a fundamental role in the baroceptor reflex integration and its action is influenced by oxidative stress.^7^

Attenuation of the baroreflex in acute kidney injury can make it difficult to respond to hemodynamic instability during an episode of acute kidney injury.^8^^,^^9^ Acute kidney injury also shows an increase in oxidative stress.^2^ Free radicals and reactive oxygen species are produced in abundance in kidney damage due to ischemia/reperfusion and flood the circulatory system causing undesirable effects on various organs, including the solitary tract, which, as we have seen, is an important integrator of the baroreflex activity.

In other models, except acute kidney injury, oxidative stress is correlated with dysfunction of the baroceptors and antioxidants improved their function. Nevertheless, in acute kidney injury, whether this increase in oxidative stress has a cause-and-effect relationship with baroreflex attenuation is controversial. Thus, if, in models of acute kidney injury, by blocking oxidative stress, we could restore the baroreflex, it would be evident that oxidative stress plays this postulated role.

The variation in heart rate to mean arterial pressure ratio compared to the challenge with phenylephrine was the baroreflex index performed in the study of Amini et al.^1^ Using a stereotactic technique, before inducing acute kidney injury, an electrode was implanted in the solitary tract nucleus of the rats with nucleus activity.^1^ Thus, in addition to improvement of the baroreflex, there was a reversal of solitary tract nucleus activity attenuation that had been documented concurrently with the reperfusion injury.

These results have pathophysiological implications, insofar as they demonstrate the participation of oxidative stress in the dysfunction of the solitary tract nucleus and consequently of the baroreflex in acute kidney injury, as well as therapeutic implications, since it encourages work with these drugs in order to mitigate the complications of acute kidney injury in humans,^9^^-^^11^ a clinical situation that denotes an ominous prognosis.^12^ Therefore, this line of research may help understand the treatment in humans with this nosological entity. It is of note that there is evidence of prophylaxis of contrast nephropathy using trimetazidine in humans.^13^^,^^14^
